# Improved Minimum Cost and Maximum Power Two Stage Genome-Wide Association Study Designs

**DOI:** 10.1371/journal.pone.0042367

**Published:** 2012-09-06

**Authors:** Stephen A. Stanhope, Andrew D. Skol

**Affiliations:** 1 Department of Human Genetics, The University of Chicago, Chicago, Illinois, United States of America; 2 Program in Genetic Medicine, The University of Chicago, Chicago, Illinois, United States of America; University of North Carolina, United States of America

## Abstract

In a two stage genome-wide association study (2S-GWAS), a sample of cases and controls is allocated into two groups, and genetic markers are analyzed sequentially with respect to these groups. For such studies, experimental design considerations have primarily focused on minimizing study cost as a function of the allocation of cases and controls to stages, subject to a constraint on the power to detect an associated marker. However, most treatments of this problem implicitly restrict the set of feasible designs to only those that allocate the same proportions of cases and controls to each stage. In this paper, we demonstrate that removing this restriction can improve the cost advantages demonstrated by previous 2S-GWAS designs by up to 40%. Additionally, we consider designs that maximize study power with respect to a cost constraint, and show that recalculated power maximizing designs can recover a substantial amount of the planned study power that might otherwise be lost if study funding is reduced. We provide open source software for calculating cost minimizing or power maximizing 2S-GWAS designs.

## Introduction

Genome-wide association studies (GWAS) have become ubiquitous in complex disease genetics. While the tools to conduct these studies have improved substantially, the cost of conducting them remains expensive. This is despite the plummeting cost per genotype, and is a result of the increasing number of markers being interrogated with each successive generation of genotyping chip. Identifying efficient study designs thus remains important.

One popular efficient design for GWAS is the two stage GWAS (2S-GWAS), which has been used for investigations of a wide range of diseases, such as type 2 diabetes [1], schizophrenia [2], lupus erythematosus [3], psoriasis [4], and breast cancer [5]. In the 2S-GWAS, a full sample of cases and controls is divided between a first stage that employs a whole genome genotyping platform and tests all available markers for association with the disease, and a second stage that uses a more expensive custom genotyping platform to follow up those markers exhibiting sufficiently strong association with the disease in stage 1. The evidence of association from both stages is then considered jointly to reach a final determination of association between marker and disease. The 2S-GWAS was shown to be more efficient than one stage analyses in which all samples are genotyped on the whole genome platform by Satagopan and Elston [6] and Thomas et al [7]. Since these early investigations, continued attention has been paid to the theoretical properties of two stage designs [8], [9], and efforts have been made to explicitly tie these theoretical properties to the problem of computing experimental designs [10]–[15]. Recent summaries of methodological and practical issues pertaining to 2S-GWAS are provided by Thomas et al [16] and Van Steen [17].

In this paper, we are concerned with computing experimental designs for 2S-GWAS. Our work is based upon that of Skol et al [10], [12], who 1) demonstrated that joint analyses that combine information on case/control allele frequency differences across stages are substantially more powerful than those based on replication, although slightly less powerful than more expensive one stage analyses; and 2) developed a software package (CaTS) to compute minimum cost designs for 2S-GWAS, subject to both an explicitly stated constraint on the minimum level of acceptable study power, and an implicitly stated equality constraint on the proportion of cases and controls allocated to the stages.

We extend this work in two ways. First, we improve the cost efficiency of the 2S-GWAS by defining a procedure that allows different proportions of cases and controls to be assigned to stages, and developing software to compute minimum cost, power constrained designs for the unrestricted 2S-GWAS. We demonstrate that the unrestricted 2S-GWAS can be substantially more cost effective than designs that restrict case and control allocation proportions to be equal. In the studies we present here, which use relatively modest differences of case and control sample sizes, we achieve up to a 40% relative cost advantage as compared to the 2S-GWAS designs computed by CaTS and 80% compared to one stage designs.

Second, and based upon our success in improving the cost effectiveness of 2S-GWAS designs relative to a power constraint, we consider 2S-GWAS designs that maximize power with respect to a cost constraint. Calculating such designs may be useful for maximizing the utility of studies that are cost constrained rather than designed to meet a given level of power, or are subject to reductions in funding relative to that required to achieve a given level of power in a cost minimizing 2S-GWAS. We examine the latter case, and demonstrate that substantial amounts of power can be retained by recalculating power maximizing experimental designs subject to cost constraints, even for large reductions in planned cost.

To support our results and assist applied researchers, our 2S-GWAS design software is included with this paper (Code S1) and available at http://www.bioinformatics.org/stanhope/2SGWASdesign/.

## Methods

### Defining a two stage GWAS with different allocations of cases and controls to stage 1

Let the total number of cases and controls be 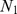 and 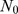 with 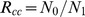 defined as the ratio of controls to cases, let 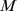 be the total number of biallelic markers to be genotyped in stage 1, and let 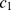 and 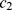 be stage 1 and stage 2 per genotype costs. We define the risk allele frequency at a hypothetical disease marker in cases and controls as 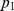 and 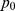 respectively, and we assume Hardy-Weinberg equilibrium within the population. Let 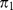 and 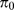 be the respective proportions of cases and controls allocated to stage 1, and let 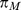 be the expected proportion of markers selected for follow-up in stage 2 if no markers are associated with disease. (Note that 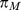 is not selected to control the type I error rate, but to reduce cost by ensuring that uninteresting markers are not genotyped in stage 2.) We suppose that the risk allele frequencies of the cases and controls assigned to stages 1 and 2 (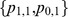 and 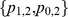 where the second term in the subscript corresponds to stage) are equal to 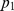 and 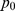 respectively. That is, there is no population heterogeneity.

**Figure 1:**
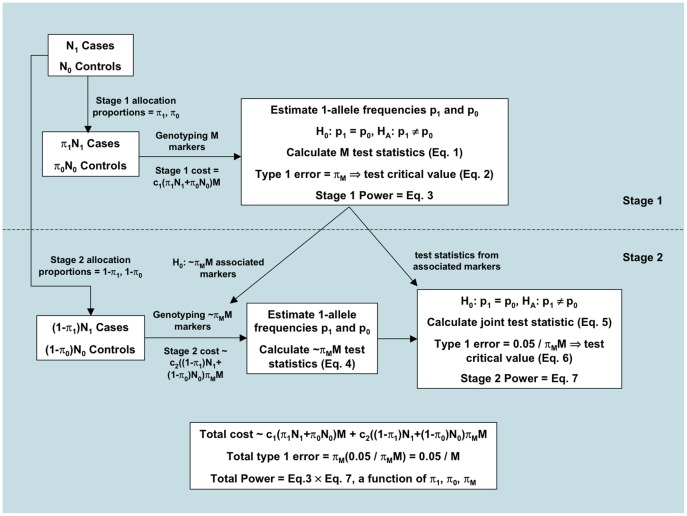
Two stage GWAS flowchart. This flowchart describes the steps of our two stage GWAS. We begin by splitting the complete data into two groups, to be used sequentially in stages 1 and 2. In the first stage, we evaluate associations of all markers with the disease. In the second stage, we genotype only those markers shown to be associated in stage 1. We compute stage 2-specific test statistics for these markers, and then construct joint test statistics based on those from both stages. The joint test statistics are used to make final assessments of disease association.

Stage 1 of the 2S-GWAS proceeds by comparing allele frequencies at each marker, using the allocated cases and controls. For each marker showing significant differences in allele frequencies between cases and controls in stage 1 (where stage 1 significance is determined by 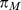), a stage 2 test of allele frequencies is calculated using the remaining cases and controls. The stage 1 and 2 test statistics are then combined according to their Fisher informations, and a joint statistic is used to evaluate the total evidence of association with the disease. For clarity, in Fig. 1 we provide a flowchart of the steps in this 2S-GWAS. The following section provides technical details. Some of the presented results have already been established (e.g. Theorem 1). However, and for clarity, we choose to err on the side of completeness.

#### The stage 1 test statistic and its asymptotic behavior

In stage 1, differences between the estimated case and control allele frequencies 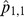 and 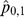 are evaluated using the statistic:
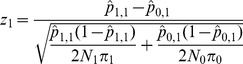
(1)Formally, we wish to evaluate 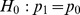 vs. 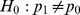 by comparing stage 1 case and control allele frequencies, and we do so by using the asymptotic distribution of 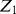.

**Theorem 1:** 
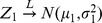
 where
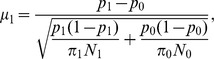


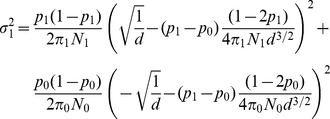
and 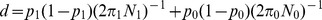.

(Proof provided in Appendix S1.)

#### Stage 1 critical value

Under 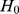, 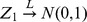 follows from Theorem 1. The critical value for the stage 1 test is therefore determined by 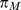, and is defined as:
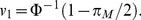
(2)Note that under the null the test is expected to pass 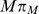 markers from stage 1 to stage 2.

#### Stage 1 power

Under the alternative, the power of the stage 1 test is:
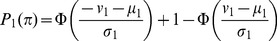
(3)where 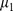 and 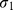 are as in Theorem 1, and we have stated 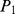 as a function of 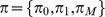.

#### The stage 2 test statistic and its asymptotic behavior

Stage 2 analysis proceeds for markers rejecting 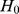 in stage 1 by estimating case and control allele frequencies based on stage 2 genotypes, 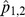 and 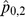, and calculating the statistic:
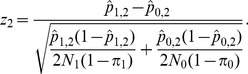
(4)The asymptotic distribution of 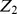 under either the null or the alternative is analogous to that of 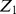.

#### Constructing the joint test statistic

After calculating 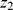, the markers under consideration are reevaluated using a joint analysis of stage 1 and stage 2 allele frequencies based on a null model Fisher information-averaged test statistic. Letting 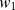 and 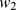 be the weights given to 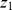 and 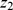, we compute the joint analysis test statistic as:
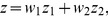
(5)where 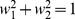, and 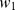 is defined:
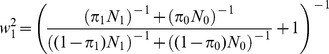
(see Appendix S1 for details).

#### Stage 2 joint test critical value calculation

Let 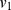 and 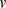 be critical values for the stage 1 and joint tests respectively. To be significantly associated with disease a marker must be rejected at both the first and second stages. Let 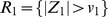 and 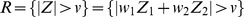 be stage 1 and 2 test rejection indicators; 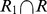 is the indicator that the marker is genome-wide significant. The probability of this event under the null can be evaluated by conditioning:
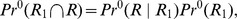
where 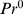 is the probability under the null model. To achieve a marker-wise type I error equal to 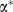, 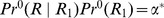 is to be maintained. 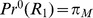 by construction, therefore 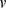 must be such that 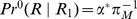.

For example, if 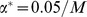 (e.g. a type I error equal to that of a Bonferroni-controlled 5% test), then 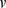 is to be set such that:
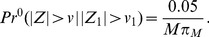
(6)We compute 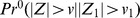 by integrating over the conditional distribution of 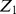 and decomposing 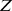 into its stage-specific portions, and numerically solve for 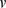 (see Appendix S1 for details).

#### Stage 2 power

At the susceptibility marker, 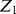 and 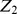 are 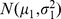 and 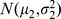 distributed (where 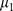 and 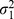 are defined in Theorem 1, and analogously for 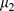 and 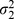). Let
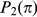
(7)be the stage 2 power stated in terms of 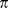. Because of its complexity, we omit providing an explicit form for 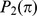 here, but do number the equation to correspond to its reference in Fig. 1. Obtaining the power of the joint test conditional on 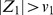 is done with a computation analogous to that used to compute type I error (see Appendix S1 for details and the explicit statement of the equation).

### Defining constrained minimum cost and maximum power two stage designs

We define an optimal two stage design as that which achieves a specified power at the least cost or, alternatively, that which maximizes power for a given experimental cost. The genotyping cost incurred when performing a 2S-GWAS is
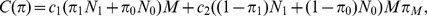
where 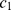 and 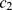 are the per marker genotyping costs for stages 1 and 2, and the total power of the 2S-GWAS is 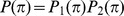. The optimal cost minimized design is that having power of at least 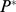 with the lowest cost 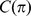. That is, the following constrained optimization problem is to be solved:
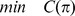


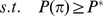
The optimization problem determining a power maximizing, cost constrained 2S-GWAS design is defined analogously:
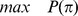


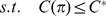



### Implementation

We developed algorithms in C to identify optimal 2S-GWAS designs as a function of 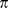. In our methods, the integration used to calculate the joint statistic's critical value and its power are computed using rectangular cubature; stage 2 critical values are obtained using the bisection method; and the three parameter constrained cost minimization and power maximization problems are solved using grid search. These algorithms are provided in Code S1.

### Evaluating the cost advantages of two stage designs allowing unequal proportions of cases and controls allocated to stage 1

We examined the cost benefits and optimal design parameters for 2S-GWAS when not restricting case-control proportions in stage 1 to be equal (i.e. 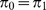) under an array of experimental conditions. Each condition was characterized by several factors that influence the optimal design and its cost: the ratio of controls to cases (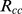); stage 2 per marker genotyping cost (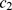, where stage 1 per marker genotyping cost is taken to be 1); disease prevalence (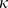); and the population frequency of the risk allele (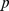). We considered studies with 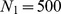 cases and 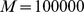 markers, and determined case and control disease allele frequencies such that a one stage GWAS would have 80% power under a multiplicative model of genetic effects with experiment-wise type I error rate of 5% and controlling for multiple testing with a Bonferroni correction using the CaTS software ([10], [12]). The full set of experimental conditions is outlined in Table S1.

For each experimental condition, we identified cost minimizing 2S-GWAS designs that would maintain 78% power both with and without the 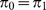 restriction, using CaTS and the unrestricted methods described in this paper respectively. (As suggested by [10] and [12], 2S-GWAS designs typically target slightly lower power levels than one stage analyses, and so we reduced our target power by 2% from the one stage baseline.) Cost minimizing designs found using the unrestricted method were determined to the nearest 1% allocation of cases and controls to stage 1 (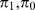) and 0.1% proportion of markers to be passed from stage 1 to stage 2 (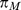). We compared the costs of the one stage and optimal 2S-GWAS designs and verified the powers of the 2S-GWAS designs and critical values computed by the unrestricted methods using 100000 sets of sampled risk allele data. Finally, we evaluated the power sensitivities of the cost minimizing 2S-GWAS designs determined by both CaTS and our unrestricted method to batch effects or genetic heterogeneity between stages by assuming the second stage case and control disease allele frequency to be 90% of that used to calculate design parameters. Using the new second stage disease allele frequency, we then re-computed the powers of the original 2S-GWAS designs and critical values using 100000 sets of sampled risk alleles. We repeated this process assuming the second stage disease allele frequency was 110% of that specified.

### Evaluating how much power is recovered by recomputing experimental designs after reductions in study funding

To examine how much power could be recovered by recomputing experimental designs after a hypothetical reduction in the funding available to a previously planned study, we focused on the set of experimental conditions defined in Table S1 with disease prevalence (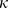) of 10% and disease allele frequency (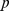) of 10%. For each experimental condition, we determined the minimum cost 78% power unrestricted 2S-GWAS design, and then calculated maximum power unrestricted 2S-GWAS designs that were constrained to cost 50%, 75% and 90% of that. We compared the maximum obtainable study power after cost constraint to the original 78% target, verified the power of the computed designs and critical values using 100000 sets of sampled risk allele data, and determined the performance sensitivity of the power maximizing designs to batch effects as we did in our analogous study in cost minimizing designs.

## Results

### Two stage designs with unequal proportions of cases and controls allocated to stage 1 are optimal when controls outnumber cases

**Figure 2:**
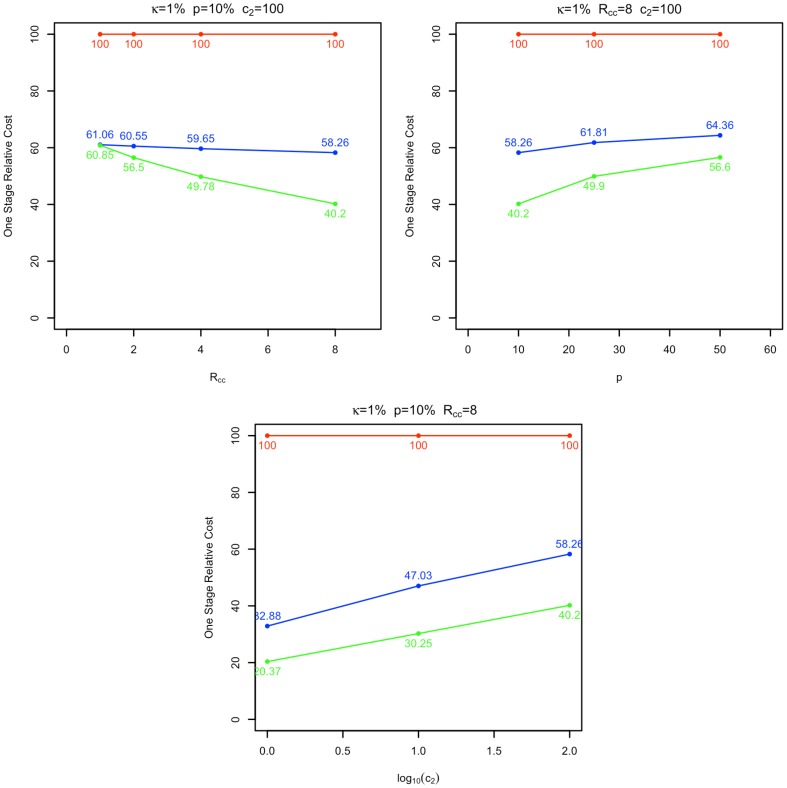
Relative cost curves for minimum cost designs. Cost curves for minimum cost, 78% power two stage GWAS designs calculated by CaTS (blue) and the unrestricted methods described here (green) relative to those of one stage GWAS designs (red) are provided for three experimental conditions: 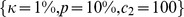 as a function of 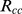; 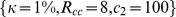 as a function of 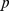; and 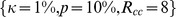 as a function of 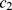 (where 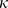 is disease prevalence, 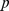 the population disease allele frequency, 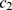 the stage 2 genotyping cost and 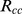 the ratio of controls to cases). Unrestricted methods show significant cost advantages in comparison to those computed by CaTS. Cost advantages increase as 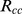 increases, 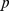 decreases, and 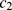 increases.

**Figure 3:**
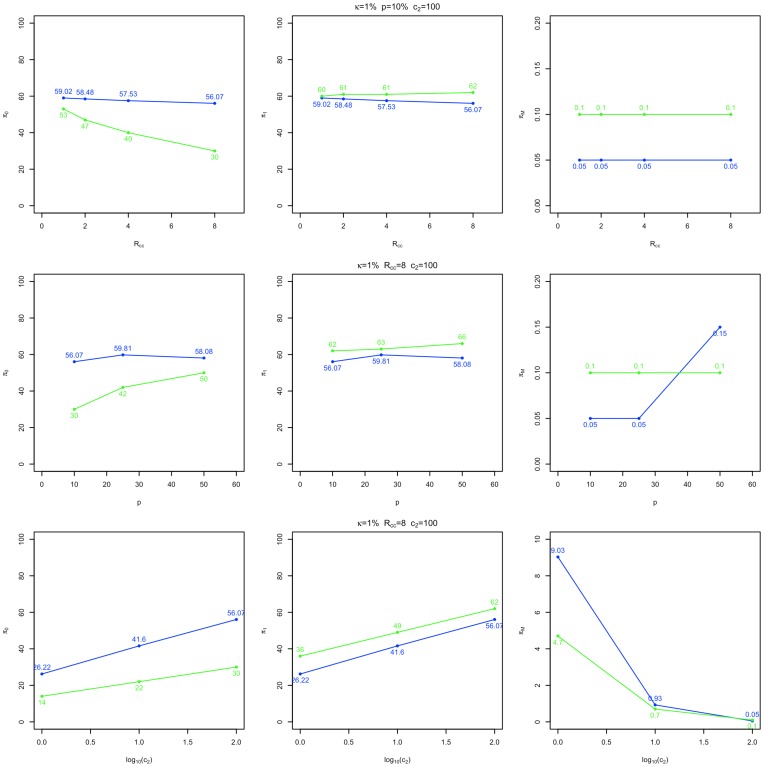
Design parameter curves for minimum cost designs. Two stage minimum cost, 78% power GWAS design parameters calculated by CaTS (blue) and the unrestricted methods described here (green) are provided for three experimental conditions: 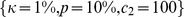 as a function of 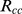; 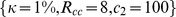 as a function of 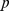; and 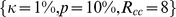 as a function of 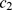 (where 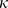 is disease prevalence, 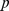 the population disease allele frequency, 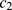 the stage 2 genotyping cost and 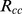 the ratio of controls to cases). Each case is assigned a row, and design parameter plots for 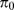, 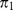 and 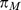 (the proportion of controls and cases assigned to stage 1, and the proportion of markers expected to be passed to stage 2) are displayed from left to right. Compared to designs computed by CaTS, designs computed without a 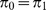 constraint typically assign lower and higher proportions of controls and cases (respectively) to stage 1, and pass a greater proportion of markers to stage 2. The degree of difference in design specification between the two methods can be substantially influenced by each of 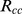, 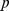 and 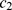.

Minimum cost experimental design parameters and performance characteristics for the full set of experimental conditions described in Table S1 are provided in Tables S2 and S3. Here, we provide plots of our results for three sets of conditions: 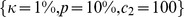, letting 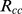 = 1, 2, 4, and 8; 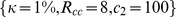, letting 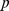 = 10, 25 and 50%; and 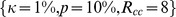 letting 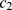 = 1, 10 and 100 (where 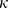 is disease prevalence, 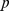 the population disease allele frequency, 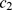 the stage 2 genotyping cost and 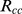 the ratio of controls to cases). Figures 2 and 3 describe the cost advantages of our unrestricted methods and characteristics of its computed design parameters respectively.

As in previous work [6], [7], two stage designs computed by both CaTS and our unrestricted algorithm have substantial cost advantages relative to the one-stage design (Fig. 2). More important from the perspective of this paper are the cost advantages of unrestricted 2S-GWAS designs relative to those computed by CaTS. This advantage increases as the ratio of controls to cases or the cost of stage 2 genotyping increases, and as population disease allele frequency decreases. For the experimental conditions plotted in Fig. 2, the unrestricted algorithm shows up to a 40% cost advantage in comparison to CaTS. These results are consistent with those provided in Tables S2 and S3, which show that although there is little difference in cost performance in 2S-GWAS designs when the number of cases equal that of controls (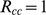), when 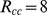 there is a 10–40% cost advantage gained by using unrestricted designs (taken across all other experimental conditions). For more modest differences between the number of cases and controls (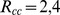), gains in cost efficiency can be observed as disease allele frequency decreases. For example, when 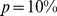 we see a cost advantage to unrestricted designs of 10–15% relative to those of CaTS.

Given the cost advantages obtained by removing the equality constraint on the proportion of controls and cases (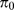 and 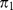 respectively) allocated to stage 1, it would be expected that optimal design parameters computed using the unrestricted procedure and CaTS will differ. For the experimental conditions plotted in Fig. 3, it can be observed that designs computed with the unrestricted algorithm assign lower and higher proportions of controls and cases (respectively) to stage 1 than those computed by CaTS, and often reduce the proportion of markers passed to stage 2 (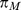). Additionally, it is clear that the degree of difference in design specification between the two methods can be influenced by each of the ratio of controls to cases, population disease allele frequency and stage 2 genotyping cost. The differences in design parameters shown in Fig. 3 are again consistent with the results presented in Tables S2 and S3.

We note that the powers of the unrestricted 2S-GWAS designs and critical values are verified in our simulation studies, with no systematic deviation from the 78% target level (Tables S2 and S3). In terms of differences between stage specific powers of the design, both unrestricted 2S-GWAS designs and those proposed by CaTS generally have higher stage 1 specific power than stage 2 power. The influence of batch effects on power were similar for experiments designed with and without the sample allocation constraint; in both types of design, scaling stage 2 case and control disease allele frequencies to 90 or 110% of those in stage 1 reduces or increases the power of a proposed design by 5–10% respectively. As the cost of stage 2 genotyping increases (holding the ratio of controls to cases constant) the sensitivity of a proposed design to batch effects is reduced.

**Figure 4:**
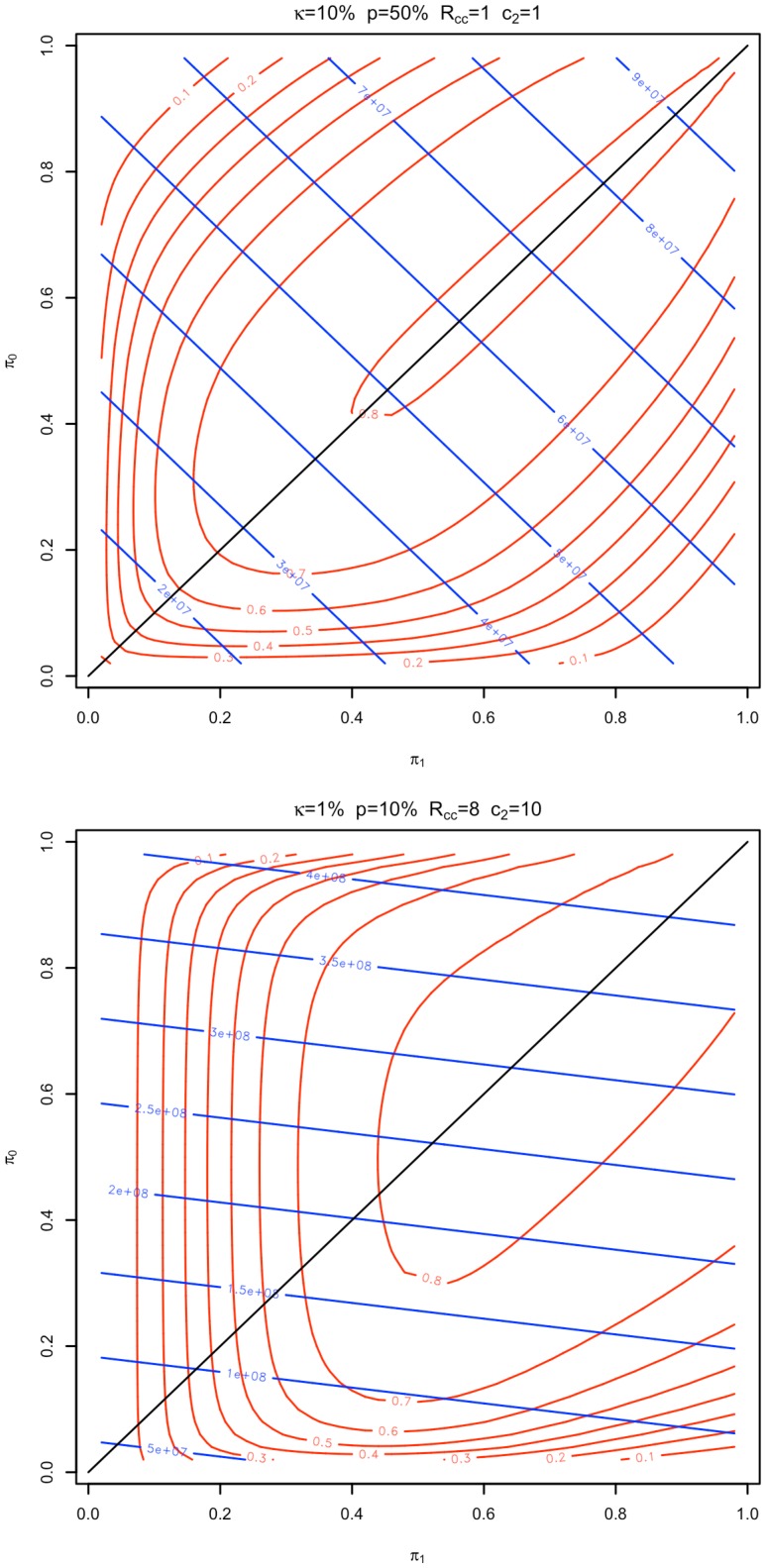
Power and cost surfaces. Power curves (red) and cost lines (blue) for the experimental conditions 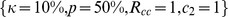 and 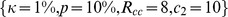 (where 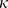 is the disease prevalence, 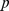 the population disease allele frequency, 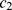 the stage 2 genotyping cost, and 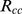 the ratio of controls to cases) are plotted as a function of the proportion of controls (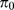) and cases (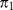) genotyped in stage 1, holding the proportion of markers followed up in stage 2 (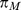) at their cost minimizing values of 8.5% and 0.7% respectively. In the first case (top), power curves and cost lines are symmetric about the identify line, implying that the cost minimizing design will use equal case and control allocations. In the second (bottom) they are asymmetric, implying that the cost minimizing design will have unequal case and control allocations.

Although it is intuitive that removing an equality constraint for the proportion of cases and controls allocated to stage 1 should improve the performance of an optimal design, it is useful to illustrate why this occurs. In Fig. 4, we plot power (red) and cost (blue) curves as functions of the proportions of controls and cases allocated to stage 1 for the experimental conditions 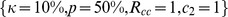, and 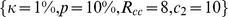, holding the expected proportion of markers to be passed to stage 2 at the cost minimizing values of 8.5% and 0.7% respectively (Tables S2 and S3). In these plots, the green identity line represents designs where the 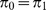 constraint holds. For the conditions with equal number of controls and cases (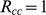) the power surface is symmetric about and has cost constrained maxima on the identity line. That is, in this case, the optimal design should have equal proportions of cases and controls allocated to stage 1. When the number of controls increases (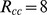), both the power surface and cost curves become asymmetric with the optimal design having 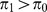.

### Power maximizing designs with unequal proportions of cases and controls allocated to stage 1 can compensate for cost reductions

**Figure 5:**
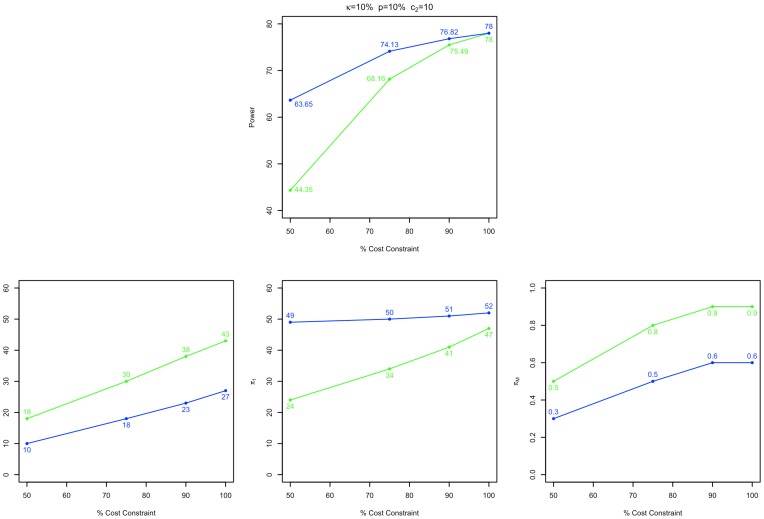
Performance and design characteristics of maximum power GWAS designs. The maximum achievable power and related experimental design parameters for the experimental condition 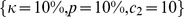 are plotted for control/case ratios 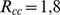 in blue and green respectively, as a function of percentage cost of a minimum cost, 78% power design. Although increasing the degree of cost constraint does have a negative effect on the achievable power of a 2S-GWAS, recomputing a power maximizing design can help to mitigate this. In comparison to the original cost minimized design, power maximizing cost limited designs typically pass a lower proportion of markers to stage 2, and then increase the relative power of the stage 2 test by allocating a greater proportion of both cases and controls to it.

Power maximizing experimental designs and their performance characteristics are provided in Table S4. In Fig. 5, we describe the results calculated for a disease with 10% prevalence (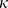), population disease allele frequency (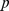) of 10%, and stage 2 genotyping cost (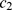) of 10, with control/case ratios (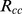) of 1 and 8 (blue and green lines respectively), as a function of the degree of relative cost restriction (50–100% of that of the cost minimizing 78% power design). As the experimental cost constraint is decreased from 100% of the minimum cost 78% power experimental design to 50% of that design, the maximum power obtainable by a cost constrained experimental design decreases from 78% to 44% and 68% for 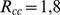 respectively. In comparison to the baseline minimum cost 78% power design, power maximizing cost limited designs typically pass a lower proportion of markers to stage 2, and then increase the relative power of the stage 2 test by allocating a greater proportion of both cases and controls to it.

The effects of diminishing experimental cost on study design and power for the experimental conditions plotted in Fig. 5 are consistent with those for the other conditions reported in Table S4. Additionally, Table S4 reports the results from validation studies of experimental power, and analyses of the sensitivity of the performance of power maximizing designs to batch effects. The levels of power calculated for the power maximizing two stage GWAS designs were verified by simulation. We observed a reduced sensitivity of the performance of power maximizing designs to batch effects as 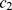 increases (holding 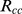 constant). This is evidenced by tightening ranges of power estimates over 90 and 110% scaling of second stage disease allele frequencies. Increases in 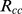 (holding 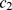 constant) did not have any such effect.

## Discussion

In many analyses of two stage genome-wide association studies (2S-GWAS) experimental design, the proportions of cases and controls allocated to stages are implicitly constrained to be the same. In this paper we have expanded the framework for 2S-GWAS originally proposed by Skol et al [10], [12] to remove this restriction. Using the expanded framework, we then demonstrated that in fact, cost-minimizing designs computed with respect to a desired level of statistical power often allocate different proportions of cases and controls to each stage. Relative to those computed under an equality constraint for proportions of cases and controls allocated to stage 1, unrestricted designs typically allocate fewer controls and more cases to stage 1, often pass fewer markers from stage 1 to stage 2, and have higher stage 1 and lower stage 2 power than those under the equality constraint. As would be expected, such designs offer substantial cost advantages relative to a 2S-GWAS that imposes equal allocation proportions. Such performance improvements become larger as the ratio of controls to cases increase, as the stage 2 per genotype cost increases, and as the population disease allele frequency decreases.

Based on this result, we extended our analysis to the problem of computing maximum power 2S-GWAS designs, subject to a cost constraint. We demonstrated that when a study budget is reduced below that of a minimum cost 2S-GWAS design meeting a targeted level of power, recomputing a maximum power 2S-GWAS design subject to the new cost constraint can retain much of the desired power. Relative to the original minimum cost design, power retention is achieved by allocating fewer cases and controls to stage 1, and passing fewer markers from stage 1 to stage 2. That is, the reduction in cost is compensated for by collecting less information in stage 1, focusing on fewer markers in stage 2, and then using a more powerful stage 2-specific test.

We note that the results achieved here are in some respects obvious - removal of a constraint from an optimization problem always weakly results in improvements in performance. However, the extent to which this is true for 2S-GWAS has not been made explicit in previous studies. Additionally, many questions pertaining to such improvements, such as why the optimal designs changed as experimental parameters changed, could only be understood by investigating the problem geometry. Related to such investigations, our studies of the sensitivity of 2S-GWAS designs to batch effects or genetic heterogeneity between stages demonstrated that our unconstrained 2S-GWAS designs are not substantially different (in that respect) from those that constrain case and control sample proportions to be equal. The gains in efficiency related to removing the sample proportion constraint do not come at the cost of higher sensitivity to batch effects.

Because most 2S-GWAS designs constrain sample allocation proportions to be the same across cases and controls, we suggest the results presented here may have implications beyond our particular study. For example, in [14], it was demonstrated that for experiments using the same numbers of cases and controls, it is in principle possible to obtain greater cost efficiencies by using three or four stages rather than two. However, the use of differential case/control allocation proportions for problems in which the numbers of cases and controls differ was not considered. Likewise, in [11], two stage GWAS designs using false discovery rate criteria were considered, again while imposing that equal numbers of cases and controls assigned to each stage. It is possible that using techniques analogous to those described here could yield greater levels of cost efficiency and power performance in such designs.

We recommend that when designing a 2S-GWAS, investigators think carefully about the relative number of controls to cases, genotyping costs, and disease allele frequencies. To assist in doing do, our programs for identifying optimal two-stage GWAS designs are provided in Code S1 or alternatively at http://www.bioinformatics.org/stanhope/2SGWASdesign/.

## Supporting Information

Appendix S1 **Supporting derivations.** This appendix provides mathematical details omitted in the main text, including the proof of Theorem 1; the Fisher information stage weighting calculation; and the stage 2 critical value and power calculations.
(PDF)

Code S1 **Supporting software.** This file contains all codes necessary to calculate cost minimizing and power maximizing two-stage GWAS designs with unequal proportions of cases and controls allocated to stages. Instructions are provided for their compilation and use, as well as example calculations.
(GZ) Click here for additional data file.

Table S1
**Experimental conditions for 2S-GWAS design calculations.** Experiments are described in terms of disease prevalences 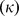; disease allele frequencies 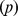; ratio of controls to cases 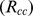; stage 2 genotyping costs 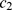 (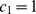 is held constant); and case/control allele frequencies 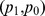. Numbers of cases and markers are constant at 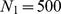 and 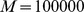.(PDF)

Table S2
**Cost minimizing 2S-GWAS designs and their performance characteristics,** 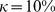 **.** For experimental conditions with 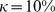 in Table S1, Table S2 reports two-stage 78% power designs computed from both CaTS and the unrestricted method. The costs of two-stage designs are compared to those of 80% power one-stage designs, and the costs of the unrestricted two-stage designs are compared to those of CaTS. Verification of the power levels of unrestricted 2S-GWAS designs is performed by Monte Carlo.(PDF)

Table S3
**Cost minimizing 2S-GWAS designs and their performance characteristics,** 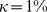 **.** For experimental conditions with 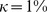 in Table S1, Table S3 reports two-stage 78% power designs computed from both CaTS and the unrestricted method. The costs of two-stage designs are compared to those of 80% power one-stage designs, and the costs of unrestricted two-stage designs are compared to those of CaTS. Verification of the power levels of unrestricted 2S-GWAS designs is performed by Monte Carlo.(PDF)

Table S4
**Power maximizing two stage GWAS designs and their performance characteristics,** 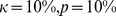 **.** For all experimental conditions with 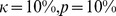 in Table S1, Table S4 reports two-stage maximum power designs and the powers they attain, with respect to a cost constraint expressed as a percentage of the cost of the minimum cost designs reported in Table S2.(PDF)
