# Supplementary material for: Improved Minimum Cost and Maximum Power Two Stage Genome-Wide Association Study Designs
Source: PLoS One. 2012 Sep 6;7(9):e42367. doi: 10.1371/journal.pone.0042367 (PMC3435377; doi:10.1371/journal.pone.0042367)
Supplement: Appendix S1 — Supporting derivations. This appendix provides mathematical details omitted in the main text, including the proof of Theorem 1; the Fisher information stage weighting calculation; and the stage 2 critical value and power calculations. (PDF) [file pone.0042367.s001.pdf]

Improved minimum cost and maximum power two-stage genome-wide association study designs: Supplementary calculation details

Stephen A. Stanhope[1], Andrew D. Skol[2]

1 Dept. of Human Genetics, The University of Chicago, Chicago, IL, USA

2 Program in Genetic Medicine, The University of Chicago, Chicago, IL, USA

∗ **E-mail: sstanhop@bsd.uchicago.edu**

Proof of Theorem 1

Without loss of generality we ignore π_1 and π_0 terms, and we assume that as $N_1 \to \infty$ the ratio of controls to cases remains $R_{cc} = N_0/N_1$. By the central limit theorem and assumed homogeneity of stage 1 and 2 samples,

$$\sqrt{N_1}(\hat{p}_{1,1} - p_1) \xrightarrow{L} N(0, p_1(1-p_1)/2),$$

$$\sqrt{N_1}(\hat{p}_{0,1} - p_0) \xrightarrow{L} N(0, p_0(1-p_0)/(2R_{cc}))$$

and the covariance between the two estimators is zero since they are based on independent samples. Next, we decompose z_1 into two parts:

$$\begin{aligned} z_1 &= \frac{\hat{p}_{1,1} - \hat{p}_{0,1}}{\sqrt{(\hat{p}_{1,1}(1-\hat{p}_{1,1})/(2N_1) + (\hat{p}_{0,1}(1-\hat{p}_{0,1}))/(2R_{cc}N_1)}} \\ &= N_1^{1/2} \frac{\hat{p}_{1,1} - \hat{p}_{0,1}}{\sqrt{(\hat{p}_{1,1}(1-\hat{p}_{1,1})/(2) + (\hat{p}_{0,1}(1-\hat{p}_{0,1}))/(2R_{cc})}} \\ &= N_1^{1/2} g(\hat{p}_{1,1}, \hat{p}_{0,1}) \end{aligned}$$

where $g(t_1, t_2)$ is now independent of N_1. The first partial derivatives of g are:

$$\frac{\partial g}{\partial t_1}(p_1, p_0) = \sqrt{\frac{1}{d}} - (p_1 - p_0)\frac{2^{-1}(1-2p_1)}{2d^{3/2}},$$

$$\frac{\partial g}{\partial t_0}(p_1, p_0) = -\sqrt{\frac{1}{d}} - (p_1 - p_0)\frac{(2R_{cc})^{-1}(1-2p_0)}{2d^{3/2}}$$

where $d = 2^{-1}p_1(1-p_1) + (2R_{cc})^{-1}p_0(1-p_0)$. We note that the first partial derivatives of g exist and are nonzero everywhere in $(0,1)^2$, so the conditions for using the delta method are met (e.g. Lehmann 1999, Chapter 5.2, Theorem 5.2.3). Therefore

$$\sqrt{N_1}(g(\hat{p}_{1,1}, \hat{p}_{0,1}) - g(p_1, p_0)) \xrightarrow{L} N(0, \tau)$$

where

$$\tau = \frac{p_1(1-p_1)}{2}\left(\frac{\partial g}{\partial t_1}(p_1,p_0)\right)^2 + \frac{p_0(1-p_0)}{2R_{cc}}\left(\frac{\partial g}{\partial t_0}(p_1,p_0)\right)^2.$$

We note that the equation given for σ_1^2 given in Theorem 1 includes the effects of the stage 1 sample allocations, and has been simplified into a more intuitive formula than that presented here.

Fisher information weight calculation

The information of a stage-specific test statistic is equal to the inverse of its variance, and the squared weight given to z_1 is therefore:

$$w_1^2 = \frac{Var\left[Z_1\right]^{-1}}{Var\left[Z_1\right]^{-1} + Var\left[Z_0\right]^{-1}}$$

where

$$Var\left[Z_1\right] = \left((2\pi_0 N_0)^{-1} + (2\pi_1 N_1)^{-1}\right) p_1\left(1-p_1\right),$$

$$Var\left[Z_0\right] = \left((2(1-\pi_0)N_0)^{-1} + (2(1-\pi_1)N_1)^{-1}\right) p_0\left(1-p_0\right).$$

The form for w_1 given in the text is by the transformation $a^{-1}\left(a^{-1}+b^{-1}\right)^{-1} = \left(ab^{-1}+1\right)^{-1}$, and is under the null of no difference between cases and controls.

Stage 2 critical value calculation details

We compute $p(v\ ; v_1) = Pr^0(|Z| > v \,|\, |Z_1| > v_1)$ by integrating over the conditional distribution of Z_1 and decomposing Z into its stage-specific portions:

$$\begin{aligned} p(v\ ; v_1) &= \int_{-\infty}^{-v_1} Pr^0(|Z| > v \mid Z_1 = z) Pr^0(Z_1 = z \mid |Z_1| > v_1) dz + \\ &\quad \int_{v_1}^{\infty} Pr^0(|Z| > v \mid Z_1 = z) Pr^0(Z_1 = z \mid |Z_1| > v_1)) dz \end{aligned}$$

where

$$\begin{aligned} Pr^0(|Z| > v \mid Z_1 = z) &= Pr^0(Z > v \mid Z_1 = z) + Pr^0(Z < -v \mid Z_1 = z) \\ &= Pr^0(w_1 Z_1 + w_2 Z_2 > v \mid Z_1 = z) + Pr^0(w_1 Z_1 + w_2 Z_2 < -v \mid Z_1 = z) \\ &= Pr^0(Z_2 > (v - w_1 z)/w_2) + Pr^0(Z_2 < (-v - w_1 z)/w_2). \end{aligned}$$

Under H_0, Z_1 and Z_2 are asymptotically $N(0,1)$ distributed, and so

$$\begin{aligned} p(v\ ; v_1) &\approx \int_{-\infty}^{-v_1} \left(\Phi\left(\frac{-v - w_1 z_1}{w_2}\right) + 1 - \Phi\left(\frac{v - w_1 z_1}{w_2}\right)\right) g(z_1\ ;\ v_1) dz_1 + \\ &\quad \int_{v_1}^{\infty} \left(\Phi\left(\frac{-v - w_1 z_1}{w_2}\right) + 1 - \Phi\left(\frac{v - w_1 z_1}{w_2}\right)\right) g(z_1\ ;\ v_1) dz_1 \end{aligned} \tag{1}$$

where

$$g(z_1; v_1) = \frac{\phi(z_1)}{\Phi(-v_1) + 1 - \Phi(v_1)}.$$

Stage 2 power calculation details

To obtain the power of the joint test conditional on $|Z_1| > v_1$ we use a computation analogous to that used to compute type I error:

$$\begin{aligned} P_2(\pi) &= \int_{-\infty}^{-v_1} h(z\ ;\ w_1, w_2, v, \mu_2, \sigma_2^2) g(z\ ;\ v_1, \mu_1, \sigma_1^2) dz + \\ & \int_{v_1}^{\infty} h(z\ ;\ w_1, w_2, v, \mu_2, \sigma_2^2) g(z\ ;\ v_1, \mu_1, \sigma_1^2) dz \end{aligned} \tag{2}$$

where:

$$\begin{aligned} h(z\ ;\ w_1, w_2, v, \mu_2, \sigma_2^2) &= \Phi\left(\frac{-(v + w_1 z)/w_2 - \mu_2}{\sigma_2}\right) + \\ & 1 - \Phi\left(\frac{(v - w_1 z)/w_2 - \mu_2}{\sigma_2}\right), \end{aligned}$$

and we define $g(z\ ;\ v_1, \mu_1, \sigma_1^2)$ analogously to what we previously defined in describing the stage 2 critical value.
