# Supplementary material for: Improved Minimum Cost and Maximum Power Two Stage Genome-Wide Association Study Designs
Source: PLoS One. 2012 Sep 6;7(9):e42367. doi: 10.1371/journal.pone.0042367 (PMC3435377; doi:10.1371/journal.pone.0042367)
Supplement: Table S1 — Experimental conditions for 2S-GWAS design calculations. Experiments are described in terms of disease prevalences ; disease allele frequencies ; ratio of controls to cases ; stage 2 genotyping costs ( is held constant); and case/control allele frequencies . Numbers of cases and markers are constant at and . (PDF) [file pone.0042367.s003.pdf]

κ (%)	p (%)	R_{cc}	c_2	p_1, p_0 (%)
10	50	1	1, 10, 100	61.7, 48.7
10	50	2	1, 10, 100	60.1, 48.9
10	50	4	1, 10, 100	59.2, 49.0
10	50	8	1, 10, 100	58.7, 49.0
10	25	1	1, 10, 100	35.7, 23.8
10	25	2	1, 10, 100	34.4, 24.0
10	25	4	1, 10, 100	33.6, 24.0
10	25	8	1, 10, 100	33.2, 24.1
10	10	1	1, 10, 100	18.0, 9.1
10	10	2	1, 10, 100	17.1, 9.2
10	10	4	1, 10, 100	16.6, 9.3
10	10	8	1, 10, 100	16.3, 9.3
1	50	1	1, 10, 100	62.8, 49.9
1	50	2	1, 10, 100	61.1, 49.9
1	50	4	1, 10, 100	60.2, 49.9
1	50	8	1, 10, 100	59.6, 49.9
1	25	1	1, 10, 100	37.0, 24.9
1	25	2	1, 10, 100	35.4, 24.9
1	25	4	1, 10, 100	34.5, 24.9
1	25	8	1, 10, 100	34.1, 24.9
1	10	1	1, 10, 100	19.1, 9.9
1	10	2	1, 10, 100	18.0, 9.9
1	10	4	1, 10, 100	17.4, 9.9
1	10	8	1, 10, 100	17.1, 9.9
