# Supplementary material for: Improved Minimum Cost and Maximum Power Two Stage Genome-Wide Association Study Designs
Source: PLoS One. 2012 Sep 6;7(9):e42367. doi: 10.1371/journal.pone.0042367 (PMC3435377; doi:10.1371/journal.pone.0042367)
Supplement: Table S2 — Cost minimizing 2S-GWAS designs and their performance characteristics, . For experimental conditions with in Table S1, Table S2 reports two-stage 78% power designs computed from both CaTS and the unrestricted method. The costs of two-stage designs are compared to those of 80% power one-stage designs, and the costs of the unrestricted two-stage designs are compared to those of CaTS. Verification of the power levels of unrestricted 2S-GWAS designs is performed by Monte Carlo. (PDF) [file pone.0042367.s004.pdf]

| Experimental Parameters | | | $p_1 = p_0$ Constrained | | | | | | Unconstrained | | |
|---|---|---|---|---|---|---|---|---|---|---|---|
| $p$ (%) | $R_{cc}$ | $c_2$ | $\pi, \pi_M$ (%) | 1S Rel cost (%) | Stage 1,2, tot. power (%) | Tot. power sensitivity | $\pi_0, \pi_1, \pi_M$ (%) | 1S, 2S Rel. cost (%) | Stage 1,2, tot. power (%) | Sim. total power (%) | Tot. power sensitivity |
| 50 | 1 | 1 | 29.66, 9.62 | 36.42 | 93.5, 83.4, 78.0 | 65.38, 87.36 | 30, 30, 8.5 | 35.95, 98.69 | 93.10, 83.79, 78.00 | 78.53 | 65.95, 87.62 |
| 50 | 1 | 10 | 44.94, 1.03 | 50.61 | 91.3, 85.4, 78.0 | 69.26, 85.16 | 44, 45, 1.0 | 50.05, 98.89 | 91.00, 85.74, 78.02 | 78.38 | 69.00, 85.22 |
| 50 | 1 | 100 | 59.59, 0.15 | 65.65 | 91.1, 85.6, 78.0 | 72.46, 84.30 | 57, 60, 0.1 | 62.65, 95.42 | 88.64, 88.0, 78.06 | 77.79 | 71.47, 82.99 |
| 50 | 2 | 1 | 31.80, 9.23 | 38.09 | 94.5, 82.5, 78.0 | 65.10, 87.73 | 31, 34, 9.3 | 38.32, 100.60 | 95.09, 82.04, 78.01 | 78.23 | 65.42, 88.16 |
| 50 | 2 | 10 | 46.26, 1.12 | 52.28 | 92.3, 84.5, 78.0 | 68.28, 85.24 | 46, 51, 0.9 | 52.37, 100.18 | 93.03, 83.85, 78.00 | 78.31 | 68.99, 85.75 |
| 50 | 2 | 100 | 61.44, 0.05 | 63.37 | 86.4, 90.3, 78.0 | 71.10, 81.40 | 59, 64, 0.1 | 64.60, 101.94 | 90.65, 86.11, 78.05 | 78.11 | 71.47, 83.37 |
| 50 | 4 | 1 | 31.79, 9.72 | 38.42 | 94.7, 82.4, 78.0 | 64.33, 86.98 | 32, 38, 9.2 | 39.34, 102.40 | 96.51, 80.82, 78.00 | 78.06 | 65.54, 88.12 |
| 50 | 4 | 10 | 48.53, 0.83 | 52.80 | 92.2, 84.6, 78.0 | 68.48, 84.82 | 46, 53, 1.1 | 53.18, 100.72 | 94.74, 82.35, 78.02 | 78.28 | 69.17, 85.83 |
| 50 | 4 | 100 | 57.79, 0.15 | 64.12 | 89.6, 87.1, 78.0 | 69.85, 82.46 | 60, 66, 0.1 | 65.08, 101.49 | 91.80, 84.99, 78.02 | 78.13 | 72.16, 83.75 |
| 50 | 8 | 1 | 31.37, 10.40 | 38.51 | 94.7, 82.4, 78.0 | 64.75, 87.56 | 27, 39, 8.9 | 34.71, 90.14 | 96.62, 80.73, 78.00 | 78.39 | 66.07, 88.22 |
| 50 | 8 | 10 | 46.32, 1.22 | 52.87 | 92.7, 84.1, 78.0 | 68.16, 84.82 | 41, 55, 0.9 | 47.72, 90.27 | 94.74, 82.34, 78.00 | 78.17 | 69.24, 85.85 |
| 50 | 8 | 100 | 57.96, 0.24 | 68.05 | 91.9, 84.9, 78.0 | 70.72, 84.30 | 53, 67, 0.1 | 59.10, 86.84 | 92.12, 84.68, 78.00 | 78.45 | 72.13, 83.81 |
| 25 | 1 | 1 | 31.00, 9.72 | 37.71 | 94.4, 82.6, 78.0 | 70.11, 84.61 | 31, 31, 9.3 | 37.41, 99.23 | 94.20, 82.81, 78.01 | 78.55 | 70.18, 84.43 |
| 25 | 1 | 10 | 47.11, 1.03 | 52.55 | 92.6, 84.2, 78.0 | 72.72, 83.48 | 44, 45, 1.3 | 51.71, 98.39 | 92.25, 84.56, 78.00 | 78.19 | 72.14, 83.13 |
| 25 | 1 | 100 | 61.13, 0.05 | 63.07 | 86.3, 90.4, 78.0 | 73.27, 80.11 | 59, 61, 0.1 | 64.00, 101.46 | 89.51, 87.21, 78.06 | 77.83 | 74.05, 81.44 |
| 25 | 2 | 1 | 29.87, 9.72 | 36.69 | 94.2, 82.8, 78.0 | 69.15, 84.27 | 28, 32, 9.1 | 35.76, 97.48 | 94.28, 82.73, 78.00 | 78.05 | 69.27, 84.38 |
| 25 | 2 | 10 | 45.37, 1.03 | 50.99 | 91.9, 84.9, 78.0 | 71.37, 82.78 | 41, 47, 1.2 | 49.84, 97.73 | 92.55, 84.31, 78.03 | 77.85 | 71.48, 83.03 |
| 25 | 2 | 100 | 60.12, 0.05 | 62.11 | 85.5, 91.2, 78.0 | 72.76, 79.39 | 55, 63, 0.1 | 61.90, 99.65 | 90.09, 86.62, 78.04 | 77.89 | 73.51, 81.75 |
| 25 | 4 | 1 | 28.36, 8.74 | 34.62 | 92.9, 84.0, 78.0 | 69.17, 84.16 | 22, 34, 7.7 | 30.22, 87.29 | 93.95, 83.04, 78.01 | 77.60 | 69.07, 84.06 |
| 25 | 4 | 10 | 42.89, 1.03 | 48.77 | 90.7, 86.0, 78.0 | 71.32, 82.44 | 35, 51, 0.8 | 43.14, 88.45 | 92.41, 84.44, 78.03 | 77.78 | 71.49, 82.64 |
| 25 | 4 | 100 | 57.71, 0.05 | 59.82 | 84.3, 92.5, 78.0 | 72.38, 79.23 | 47, 62, 0.1 | 55.00, 91.93 | 89.59, 87.14, 78.07 | 77.70 | 73.57, 81.79 |
| 25 | 8 | 1 | 29.65, 9.03 | 36.00 | 94.0, 83.0, 78.0 | 68.34, 84.11 | 21, 36, 8.3 | 29.08, 80.78 | 95.80, 81.42, 78.00 | 77.82 | 68.55, 84.43 |
| 25 | 8 | 10 | 44.05, 1.12 | 50.31 | 91.9, 84.9, 78.0 | 70.79, 82.83 | 35, 53, 0.7 | 41.41, 82.29 | 93.60, 83.35, 78.02 | 77.64 | 71.48, 82.86 |
| 25 | 8 | 100 | 59.47, 0.05 | 61.49 | 85.6, 91.1, 78.0 | 71.78, 79.23 | 45, 65, 0.1 | 52.50, 85.37 | 91.71, 85.08, 78.03 | 77.37 | 73.14, 81.51 |
| 10 | 1 | 1 | 30.47, 10.21 | 37.56 | 94.6, 82.5, 78.0 | 71.74, 84.03 | 29, 29, 9.5 | 35.74, 95.14 | 93.45, 83.47, 78.00 | 78.20 | 71.34, 83.18 |
| 10 | 1 | 10 | 46.32, 1.03 | 51.84 | 92.4, 84.4, 78.0 | 73.71, 82.67 | 43, 47, 0.9 | 49.95, 96.33 | 91.17, 85.57, 78.02 | 77.97 | 73.31, 81.87 |
| 10 | 1 | 100 | 61.03, 0.05 | 62.97 | 86.4, 90.3, 78.0 | 74.43, 79.88 | 56, 60, 0.1 | 62.20, 98.76 | 88.70, 87.98, 78.03 | 78.30 | 75.12, 80.69 |
| 10 | 2 | 1 | 28.86, 9.72 | 35.77 | 94.3, 82.7, 78.0 | 71.15, 83.92 | 22, 29, 9.4 | 31.44, 87.89 | 92.59, 84.27, 78.00 | 77.62 | 70.07, 82.99 |
| 10 | 2 | 10 | 45.06, 0.93 | 50.16 | 91.9, 84.9, 78.0 | 73.06, 82.69 | 36, 46, 0.9 | 44.79, 89.28 | 90.52, 86.21, 78.03 | 77.55 | 72.16, 81.60 |
| 10 | 2 | 100 | 59.37, 0.15 | 65.46 | 91.9, 84.9, 78.0 | 75.54, 82.86 | 48, 60, 0.1 | 56.80, 86.76 | 88.31, 88.34, 78.01 | 77.61 | 74.03, 80.60 |
| 10 | 4 | 1 | 27.92, 9.91 | 33.28 | 92.9, 84.0, 78.0 | 69.79, 83.64 | 21, 33, 7.2 | 28.92, 86.88 | 97.87, 79.71, 78.00 | 77.00 | 69.59, 83.66 |
| 10 | 4 | 10 | 43.67, 1.12 | 47.52 | 90.6, 86.1, 78.0 | 71.81, 82.22 | 34, 50, 0.7 | 41.60, 87.54 | 95.81, 81.42, 78.01 | 77.27 | 71.27, 81.93 |
| 10 | 4 | 100 | 58.73, 0.05 | 59.49 | 87.4, 89.2, 78.0 | 73.10, 79.01 | 44, 64, 0.1 | 53.20, 89.43 | 93.01, 83.78, 78.01 | 77.23 | 72.83, 80.39 |
| 10 | 8 | 1 | 25.78, 9.52 | 32.84 | 92.9, 84.0, 78.0 | 69.11, 82.51 | 16, 35, 6.2 | 23.18, 70.59 | 94.54, 82.51, 78.01 | 76.67 | 69.26, 82.71 |
| 10 | 8 | 10 | 39.41, 1.42 | 48.01 | 91.3, 85.4, 78.0 | 71.12, 81.99 | 27, 52, 0.6 | 33.99, 70.79 | 92.96, 83.94, 78.03 | 77.33 | 71.56, 81.19 |
| 10 | 8 | 100 | 56.14, 0.15 | 62.71 | 90.0, 85.8, 78.0 | 73.88, 81.66 | 36, 61, 0.1 | 44.90, 71.58 | 90.10, 86.62, 78.04 | 77.13 | 72.95, 80.30 |
