# Supplementary material for: Improved Minimum Cost and Maximum Power Two Stage Genome-Wide Association Study Designs
Source: PLoS One. 2012 Sep 6;7(9):e42367. doi: 10.1371/journal.pone.0042367 (PMC3435377; doi:10.1371/journal.pone.0042367)
Supplement: Table S3 — Cost minimizing 2S-GWAS designs and their performance characteristics, . For experimental conditions with in Table S1, Table S3 reports two-stage 78% power designs computed from both CaTS and the unrestricted method. The costs of two-stage designs are compared to those of 80% power one-stage designs, and the costs of unrestricted two-stage designs are compared to those of CaTS. Verification of the power levels of unrestricted 2S-GWAS designs is performed by Monte Carlo. (PDF) [file pone.0042367.s005.pdf]

Experimental Parameters			$p_1 = p_0$ Constrained				Unconstrained				
p (%)	R_{cc}	c_2	π, π_M (%)	1S Rel cost (%)	Stage 1,2, tot. power (%)	Tot. power sensitivity	π_0, π_1, π_M (%)	1S, 2S Rel. cost (%)	Stage 1,2, tot. power (%)	Sim. total power (%)	Tot. power sensitivity
50	1	1	31.38, 10.21	38.39	94.8, 82.3, 78.0	64.98, 88.03	32, 31, 9.9	38.28, 99.73	94.70, 82.37, 78.00	78.32	65.27, 88.11
50	1	10	46.21, 1.03	52.23	92.7, 84.1, 78.0	68.64, 85.42	46, 46, 1.2	52.48, 100.47	92.62, 84.21, 78.00	78.05	68.52, 85.97
50	1	100	58.84, 0.15	65.45	89.7, 87.0, 78.0	71.25, 84.00	60, 62, 0.1	64.90, 99.16	89.96, 86.74, 78.04	78.55	72.04, 84.00
50	2	1	30.48, 10.11	37.50	94.2, 82.8, 78.0	65.01, 87.54	29, 33, 9.3	36.81, 98.14	94.41, 82.64, 78.02	78.34	65.40, 88.22
50	2	10	45.06, 1.22	51.76	92.1, 84.7, 78.0	68.22, 85.51	44, 50, 0.9	50.86, 98.25	92.36, 84.48, 78.02	78.18	68.87, 85.81
50	2	100	60.76, 0.05	62.72	86.0, 90.7, 78.0	70.90, 80.90	57, 63, 0.1	63.10, 100.60	89.96, 86.77, 78.06	77.86	72.55, 83.89
50	4	1	29.66, 9.33	36.22	93.3, 83.6, 78.0	65.66, 87.83	23, 34, 8.1	31.25, 86.29	93.55, 83.38, 78.01	78.30	65.49, 87.12
50	4	10	44.09, 1.12	50.35	91.1, 85.6, 78.0	69.01, 85.63	36, 50, 0.9	44.30, 87.99	91.82, 84.97, 78.02	78.24	69.03, 85.67
50	4	100	59.27, 0.15	65.37	90.9, 85.8, 78.0	72.80, 85.06	48, 64, 0.1	56.08, 85.77	89.89, 86.82, 78.04	78.38	71.77, 83.85
50	8	1	31.78, 10.01	38.60	94.7, 82.4, 78.0	65.07, 87.94	25, 39, 7.9	32.35, 83.80	96.06, 81.20, 78.00	78.20	65.49, 88.24
50	8	10	46.97, 1.12	52.90	92.6, 84.2, 78.0	68.68, 85.71	38, 54, 0.9	45.19, 85.42	94.21, 82.82, 78.02	78.19	69.22, 86.06
50	8	100	58.08, 0.15	64.36	89.7, 87.0, 78.0	70.81, 83.13	50, 66, 0.1	56.60, 87.93	91.53, 85.23, 78.02	78.38	72.08, 83.84
25	1	1	28.79, 9.62	35.64	93.0, 83.9, 78.0	70.23, 84.79	28, 29, 9.3	35.14, 98.63	95.00, 82.12, 78.01	77.97	69.79, 84.24
25	1	10	42.85, 1.22	49.82	90.9, 85.8, 78.0	71.83, 82.51	43, 44, 1.0	49.15, 98.65	92.72, 84.15, 78.02	77.17	71.37, 82.05
25	1	100	58.59, 0.05	60.66	84.6, 92.2, 78.0	72.89, 79.42	55, 60, 0.1	61.75, 101.79	89.96, 86.74, 78.04	78.13	73.77, 81.27
25	2	1	30.97, 9.03	37.20	94.4, 82.6, 78.0	69.61, 84.41	27, 32, 9.3	35.30, 94.88	94.16, 82.84, 78.00	77.89	69.39, 84.50
25	2	10	45.39, 1.12	51.50	92.3, 84.5, 78.0	71.87, 83.09	41, 47, 1.1	49.27, 95.65	92.13, 84.69, 78.02	77.98	71.18, 82.76
25	2	100	60.70, 0.05	62.66	86.2, 90.5, 78.0	73.13, 80.15	57, 63, 0.1	63.10, 100.69	89.96, 86.77, 78.06	78.14	74.07, 82.13
25	4	1	30.06, 10.11	37.13	94.7, 82.4, 78.0	68.35, 84.31	27, 35, 8.9	34.95, 94.13	95.69, 81.53, 78.01	77.71	68.83, 84.60
25	4	10	45.38, 1.12	51.49	92.5, 84.3, 78.0	70.66, 82.67	41, 50, 1.0	48.52, 94.21	93.59, 83.35, 78.01	77.46	70.79, 83.05
25	4	100	60.74, 0.05	62.70	86.4, 90.3, 78.0	72.47, 79.58	48, 64, 0.1	56.08, 89.43	89.89, 86.82, 78.04	76.72	72.10, 80.75
25	8	1	29.67, 9.42	36.29	94.2, 82.8, 78.0	69.06, 84.96	20, 37, 6.4	26.88, 74.08	94.93, 82.17, 78.00	77.83	68.86, 84.36
25	8	10	44.97, 1.12	51.13	92.5, 84.3, 78.0	71.43, 83.38	31, 52, 0.8	38.66, 75.61	93.39, 83.55, 78.02	77.78	71.25, 82.88
25	8	100	59.81, 0.05	61.81	85.8, 90.9, 78.0	73.11, 80.17	42, 63, 0.1	49.90, 88.16	90.52, 86.19, 78.02	77.81	72.89, 81.24
10	1	1	26.86, 9.81	34.03	93.5, 83.4, 78.0	71.17, 83.02	27, 27, 9.9	34.22, 100.56	92.34, 84.47, 78.00	78.30	71.61, 83.23
10	1	10	43.73, 1.12	50.03	91.3, 85.4, 78.0	73.96, 82.73	42, 44, 0.9	48.13, 96.19	89.89, 86.78, 78.00	78.05	72.90, 81.68
10	1	100	59.02, 0.05	61.06	85.0, 91.8, 78.0	74.46, 79.53	53, 60, 0.1	60.85, 99.64	87.93, 88.78, 78.06	78.23	74.91, 80.87
10	2	1	27.92, 9.91	35.06	93.8, 83.2, 78.0	71.20, 83.95	22, 30, 8.6	31.14, 88.82	92.38, 84.44, 78.01	77.71	70.15, 83.00
10	2	10	44.17, 1.03	49.92	91.9, 84.9, 78.0	73.37, 82.98	35, 47, 0.9	44.49, 89.12	90.65, 86.06, 78.01	77.47	72.27, 81.85
10	2	100	58.48, 0.05	60.55	85.0, 91.8, 78.0	73.70, 79.55	47, 61, 0.1	56.50, 93.30	88.52, 88.12, 78.00	77.47	73.77, 80.53
10	4	1	27.55, 9.13	34.16	93.6, 83.3, 78.0	70.65, 83.93	18, 32, 7.2	26.50, 77.57	92.78, 84.09, 78.02	77.40	69.78, 82.78
10	4	10	41.91, 1.22	48.99	91.8, 85.0, 78.0	73.05, 83.21	30, 49, 0.7	38.43, 78.44	91.06, 85.66, 78.00	77.13	71.59, 81.50
10	4	100	57.53, 0.05	59.65	84.5, 92.3, 78.0	73.62, 79.28	40, 61, 0.1	49.78, 83.44	89.10, 87.57, 78.03	77.42	72.92, 80.36
10	8	1	26.22, 9.03	32.88	92.8, 84.1, 78.0	70.65, 83.88	14, 36, 4.7	20.37, 61.95	93.04, 83.85, 78.02	77.09	70.00, 82.68
10	8	10	41.60, 0.93	47.03	90.3, 86.4, 78.0	73.12, 83.21	22, 49, 0.7	30.25, 64.31	91.43, 85.34, 78.03	77.00	71.13, 81.32
10	8	100	56.07, 0.05	58.26	83.4, 93.6, 78.0	73.37, 78.69	30, 62, 0.1	40.20, 68.99	90.05, 86.64, 78.03	77.27	72.97, 80.50
