# Supplementary material for: Improved Minimum Cost and Maximum Power Two Stage Genome-Wide Association Study Designs
Source: PLoS One. 2012 Sep 6;7(9):e42367. doi: 10.1371/journal.pone.0042367 (PMC3435377; doi:10.1371/journal.pone.0042367)
Supplement: Table S4 — Power maximizing two stage GWAS designs and their performance characteristics, . For all experimental conditions with in Table S1, Table S4 reports two-stage maximum power designs and the powers they attain, with respect to a cost constraint expressed as a percentage of the cost of the minimum cost designs reported in Table S2. (PDF) [file pone.0042367.s006.pdf]

Experimental Parameters						
R_{cc}	c_2	% min cost	π_0, π_1, π_M (%)	Stage 1,2, tot. power (%)	Sim. total power (%)	Tot. power sensitivity
1	1	90	25, 26, 8.9	89.97, 84.57, 76.08	76.41	69.46, 81.07
1	1	75	20, 22, 7.3	81.91, 86.58, 70.91	71.03	65.79, 76.46
1	1	50	12, 14, 5.6	58.75, 90.27, 53.03	53.98	50.08, 56.64
2	1	90	19, 28, 8.0	89.28, 85.08, 75.95	75.54	68.33, 80.64
2	1	75	15, 25, 6.4	81.51, 86.90, 70.83	70.05	63.68, 74.83
2	1	50	8, 18, 4.9	58.57, 90.57, 53.04	53.11	49.10, 55.92
4	1	90	18, 33, 6.3	91.54, 81.23, 74.36	73.52	68.14, 81.40
4	1	75	13, 30, 6.3	86.55, 82.04, 71.00	70.39	64.96, 77.80
4	1	50	7, 26, 4.1	67.94, 85.41, 58.02	57.52	54.06, 63.52
8	1	90	14, 35, 5.4	93.11, 82.63, 76.93	75.85	68.00, 81.78
8	1	75	11, 35, 4.3	89.91, 82.82, 74.46	73.57	65.80, 79.07
8	1	50	6, 34, 2.7	77.08, 83.94, 64.70	64.00	57.57, 68.88
1	10	90	38, 41, 0.9	86.45, 87.32, 75.49	75.88	70.98, 79.30
1	10	75	30, 34, 0.8	75.58, 90.19, 68.16	68.55	64.24, 71.04
1	10	50	18, 24, 0.5	46.76, 94.86, 44.35	44.85	42.98, 46.29
2	10	90	30, 43, 0.9	86.39, 87.30, 75.42	75.21	70.00, 79.10
2	10	75	23, 42, 0.6	77.17, 89.16, 68.80	68.81	64.41, 72.06
2	10	50	13, 31, 0.4	48.49, 94.51, 45.83	45.90	43.73, 47.52
4	10	90	29, 47, 0.7	88.25, 83.93, 74.06	73.37	69.59, 79.91
4	10	75	22, 46, 0.6	82.97, 84.57, 70.16	69.62	66.32, 75.73
4	10	50	11, 39, 0.5	61.50, 88.41, 54.37	54.18	52.08, 59.01
8	10	90	23, 51, 0.6	91.53, 83.94, 76.82	75.68	69.82, 80.28
8	10	75	18, 50, 0.5	87.84, 84.41, 74.13	73.05	67.61, 77.55
8	10	50	10, 49, 0.3	74.34, 85.62, 63.65	63.24	58.22, 66.80
1	100	90	49, 53, 0.1	82.41, 90.42, 74.51	74.64	71.40, 77.10
1	100	75	37, 44, 0.1	68.43, 93.74, 64.14	64.94	61.75, 65.51
1	100	50	19, 27, 0.1	33.60, 97.60, 32.79	32.86	32.22, 33.10
2	100	90	40, 57, 0.1	83.50, 89.61, 74.82	74.89	71.12, 77.68
2	100	75	28, 52, 0.1	71.88, 91.70, 65.91	65.78	62.05, 68.52
2	100	50	13, 35, 0.1	36.74, 96.94, 35.61	36.02	34.83, 36.64
4	100	90	37, 62, 0.1	87.17, 84.57, 73.72	72.88	71.03, 78.92
4	100	75	27, 58, 0.1	79.79, 86.10, 68.70	68.54	66.53, 73.91
4	100	50	11, 48, 0.1	50.45, 90.77, 45.79	45.97	45.16, 49.71
8	100	90	30, 63, 0.1	90.07, 85.11, 76.66	75.67	71.00, 79.25
8	100	75	22, 60, 0.1	85.29, 85.84, 73.21	72.49	68.14, 75.83
8	100	50	9, 52, 0.1	62.88, 88.84, 55.85	55.31	51.86, 58.21
